# Supplementary material for: Trifuhalol A Suppresses Allergic Inflammation through Dual Inhibition of TAK1 and MK2 Mediated by IgE and IL-33
Source: Int J Mol Sci. 2022 Sep 5;23(17):10163. doi: 10.3390/ijms231710163 (PMC9456157; doi:10.3390/ijms231710163)
Supplement: Supplementary file 1 [file ijms-23-10163-s001.zip › ijms-1870782-supplementary.pdf]

Supplementary data

Trifuhalol A suppresses allergic inflammation through dual inhibition of TAK1 and MK2 mediated by IgE and IL-33

Sim-Kyu Bong[1], No-June Park[1,2], Sang Heon Lee[1], Jin Woo Lee[1], Aaron Taehwan Kim[3], Xiaoyong Liu[4], Sang Moo Kim[5], Min Hye Yang[6], Yong Kee Kim[7,*], Su-Nam Kim[1,2,*]

[1] Natural Products Research Institute, Korea Institute of Science and Technology, 679 Saimdang-ro, Gangneung, Gangwon-do 25451, Republic of Korea.

[2] Division of Bio-Medical Science and Technology, KIST School, University of Science and Technology, Seoul, 02792, Republic of Korea

[3] Department of Food Science, University of Massachusetts, Amherst, MA, USA

[4] Haizhibao Deutschland GmbH, Heiliggeistgasse 28, 85354 Freising Munish, Germany.

[5] Department of Marine Food Science and Technology, Gangneung-Wonju National University, 7 Jukheon-gil, Gangneung 25457, Republic of Korea

[6] College of Pharmacy, Pusan National University, Busan 46241, Republic of Korea

[7] College of Pharmacy, Sookmyung Women's University, Seoul 04310, Republic of Korea

***Corresponding author:** Yong Kee Kim, Ph.D., College of Pharmacy, Sookmyung Women's University, 100 Cheongpa-ro 47-gil, Yongsan-gu, Seoul 04610, Republic of Korea, Tel: +82-2-2077-7688, Fax: +82-2-710-9871, E-mail: yksnbk@sookmyung.ac.kr; Su-Nam Kim, Ph.D., Natural Products Research Institute, Korea Institute of Science and Technology, 679 Saimdang-ro, Gangneung, Gangwon-do 25451, Republic of Korea, Tel: +82-33-650-3503, Fax: +82-33-650-3529, E-mail: snkim@kist.re.kr

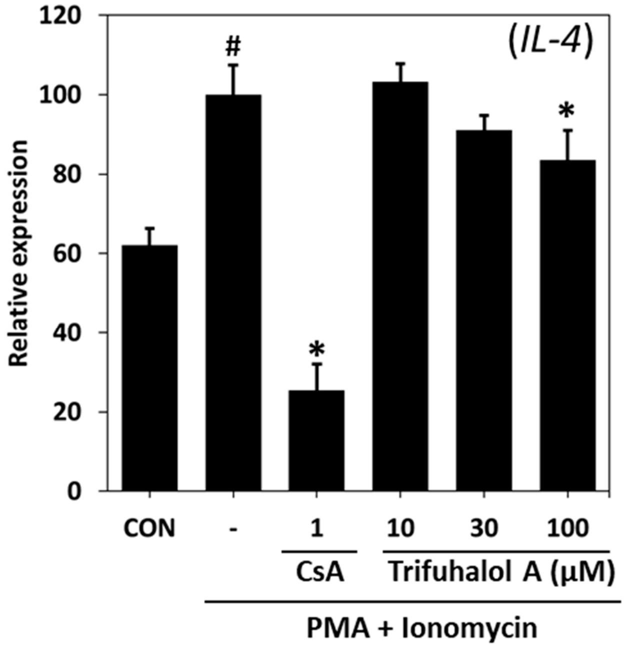

Supplementary Figure S1. Effects of trifuhalol A on IL-4 production. Representative data of qPCR analysis of IL-4 mRNA normalized to GAPDH in PMA/ionomycin (PI)-induced RBL-2H3 cells. RBL-2H3 cells were treated with trifuhalol A in combination with PMA (50 μg/ml)/ionomycin (0.1 μM) for 8 h to measure IL-4 expression. Each bar represents the mean ± S.D. of duplicates. #P < 0.05 vs. control; *P < 0.05 vs. PI-induced group. CsA; 1μM of cyclosporine A.
